# Different multi-year mean temperature in mid-summer of South China under different 1.5 °C warming scenarios

**DOI:** 10.1038/s41598-018-32277-6

**Published:** 2018-09-14

**Authors:** Xia Qu, Gang Huang

**Affiliations:** 1 State Key Laboratory of Numerical Modeling for Atmospheric Sciences and Geophysical Fluid Dynamics, Institute of Atmospheric Physics, Chinese Academy of Sciences, Beijing, 100029 China; 2 Laboratory for Regional Oceanography and Numerical Modeling, Qingdao National Laboratory for Marine Science and Technolog, Qingdao, 266237 China; 3 University of Chinese Academy of Sciences, Beijing, 100049 China

## Abstract

The Paris Agreement proposed a goal of “pursuing efforts to limit the temperature increase to 1.5 °C above pre-industrial levels”. The Community Earth System Model, version 1, with the Community Atmosphere Model, version 5 (CESM1-CAM5), designed a set of experiments that fulfilled the 1.5 °C warming goal. By analyzing the outputs, this study aims to present projections associated with warming in South China (SC). Interestingly, if the global mean temperature (GMT) overshoots to 1.7 °C above the pre-industrial levels in 2050 and back to 1.5 °C by 2100, additional warming in the SC mid-summer will occur when approaching 2100 compared to that in the scenario under which the GMT stabilizes at an increase of 1.5 °C after the mid-2040 s. In the final 1 to 3 decades of 21^st^ century in most parts of SC, the multi-year mean warming differences, as well as the difference of extreme hot days, between the two scenarios are significant among the ensembles in mid-summer. Under the scenario in which the GMT overshoots an increase of 1.5 °C, the decrease of mid-level clouds leads to increased downwards solar radiation in the SC and warms the surface, resulting in increases in both outgoing longwave radiation and latent heat flux into the atmosphere and maintenance of the surface balance of the heat budget.

## Introduction

In 2015, the Paris Agreement proposed the target of “Holding the increase in the global average temperature to well below 2 °C above pre-industrial levels and pursuing efforts to limit the temperature increase to 1.5 °C above pre-industrial levels”^1^. To achieve the 1.5 °C warming target, the concentration of greenhouse gases (GHGs) should decline before 2060; while the concentration of GHGs needs to decrease before 2085 to realize the 2 °C warming target^2^. With the drop of the concentration of GHGs concentration, the global temperature will exhibit a warming inertia^3,4^, mainly due to the large heat storage capacity of the deep ocean^3^. The role of the deep ocean during the drop of the GHG concentration is quite different to that when the concentration increases, which leads to a very different ocean surface warming pattern and a different climate response of the atmosphere^4,5^. Thus, the climate risk under the 1.5 °C warming scenarios may not be able to be linearly predicted from the results under the Representative Concentration Pathway (RCP) 8.5 scenarios, under which the concentration of GHGs does not decline. The RCP2.6 experiment is quite similar to the 1.5 °C warming scenario. Despite the concentration of GHGs decreasing after 2046, the RCP2.6 experiment may not be strictly appropriate for a 1.5 °C study because its multi-model mean GMT is above 1.5 °C in 2100^6^.

This study shows that the different trajectories of GHG radiation for reaching 2 °C warming lead to different regional precipitation responses^7^. Thus, there is the possibility that the different way in which the 1.5 °C warming goal is reached may lead to different responses in the regional climate.

Recently, a “low warming” experiment was conducted by CESM1-CAM5. This experiment included one set of simulations whose GMT increase was 1.5 °C in 2100 relative to the pre-industrial levels. CESM1-CAM5 consists of experiments in which the GMT increase never exceeds 1.5 °C (hereafter the NE scenario) and the increase slightly overshoots 1.5 °C (hereafter OS scenario)^8^. The simulation mainly focuses on the climate change in the 1.5 NE scenario and compared it with the changes in a 2.0 °C warming world. The study laid quite low stress on the differences between the NE and OS scenarios in the 1.5 °C warming climate.

In Asia, the SC is one of the most populated regions. Boreal summer is the hottest season in the SC, with temperature peaks in July. The SC frequently suffers extremely hot events^9–12^, which lead to large social and economic losses. Here, we find that that are differences in the multi-year mean temperature in the SC mid-summer under different emission trajectories for achieving the Paris Agreement 1.5 °C warming goal.

## Results

First, the reproducibility of CESM1-CAM5 on the SC summer temperature is evaluated based on the *S*-index, which is described by equation (1) in the methods section. For the period 1976–2005, the *S*-indices of the July temperature are 0.837 to 0.843. These indices are quite close to 1.0, indicating the good reproducibility of CESM1-CAM5 for the summer temperature in the SC.

**Figure 1:**
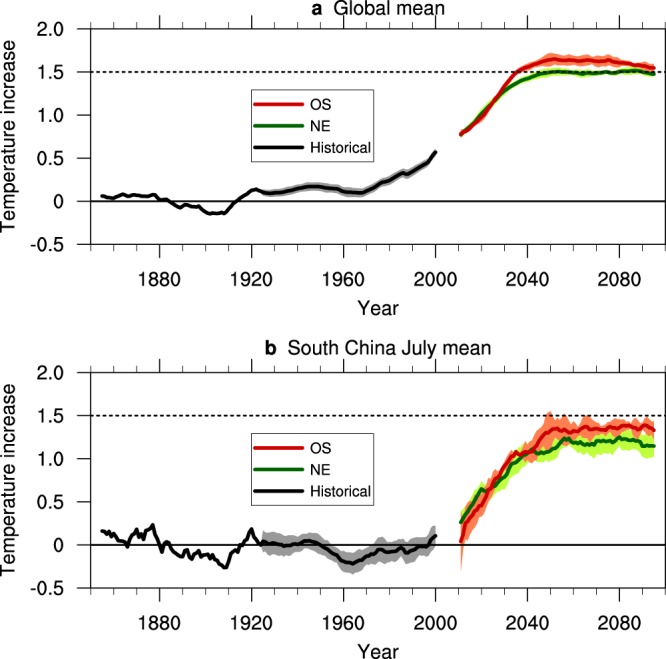
Temperature increases relative to the pre-industrial level. (**a**) Global mean temperature increases in CESM-CAM5; and (**b**) July mean temperature increase in South China in CESM-CAM5. The unit is °C. The solid lines indicate the temperature increase. The shadings indicate the standard deviation of the ensembles. The temperature in each figure was subtracted by its average from 1850 to 1920, and 11-point smoothening was performed on the results. The black, green and red lines indicate the Large Ensemble, NE and OS simulations, respectively. The results during 1850–1920 are the results of member “001”; the results during 1920–2005 are the mean of the 35 members of the CESM-CAM5 Large Ensemble experiment; and the results of the NE scenario are the mean of the 10 members from the NE experiment, and those of the OS scenario are the means of the 5 members from the OS experiment.

In 2005, the GMT increase relative to the pre-industrial level (1850–1920) was approximately 0.6 °C (Fig. 1a). At the end of the 21^st^ century, the GMT under both 1.5 °C warming scenarios increased to 0.9 °C above the 2005 level, reaching 1.5 °C warming. The differences under the two scenarios are: (1) GMT stabilizes at 1.5 °C warming after the mid-2040s under the NE scenario; (2) GMT under the OS scenario reaches 1.7 °C warming in 2050 and then fluctuates over the next 2–3 decades and drops to 1.5 °C warming in 2100. When approaching the end of the 21^st^ century, the difference between the NE and OS scenarios are reduced. The GMT from 1850 to 1920 displays fluctuations relative to other periods because CESM1-CAM5 only has one ensemble during this period and the results during 1920–2005 are a 35-ensemble mean.

Interestingly, we find that the multi-year mean of the SC mid-summer temperature is different between the NE and OS before 2100. The July temperature in the SC shows a more intensive interannual fluctuation than the GMT (Fig. 1b), mainly because the SC temperature is subjected to other factors besides carbon emission, for instance, El Niño and South Oscillation^13^. At the end of the 21^st^ century, the July temperature increase relative to the pre-industrial level in the SC is less than that of the GMT under both the NE and OS scenarios. The July temperature increase in the SC in 2005 is approximately 0.25 °C. Under the NE scenario, relative to 2005, the increase in 2100 is 0.86 °C, with a 2091–2100 mean increase of 0.90 °C; under the OS scenario, the increases are 0.91 °C and 1.08 °C, respectively. Unlike the GMT, when approaching the end of the 21^st^ century, no evidence indicates that the gap between the NE and OS scenarios will narrow. Therefore, though both experiments achieve the 1.5 °C warming goal, SC will surfer a warmer mid-summer under the OS scenario.

**Figure 2:**
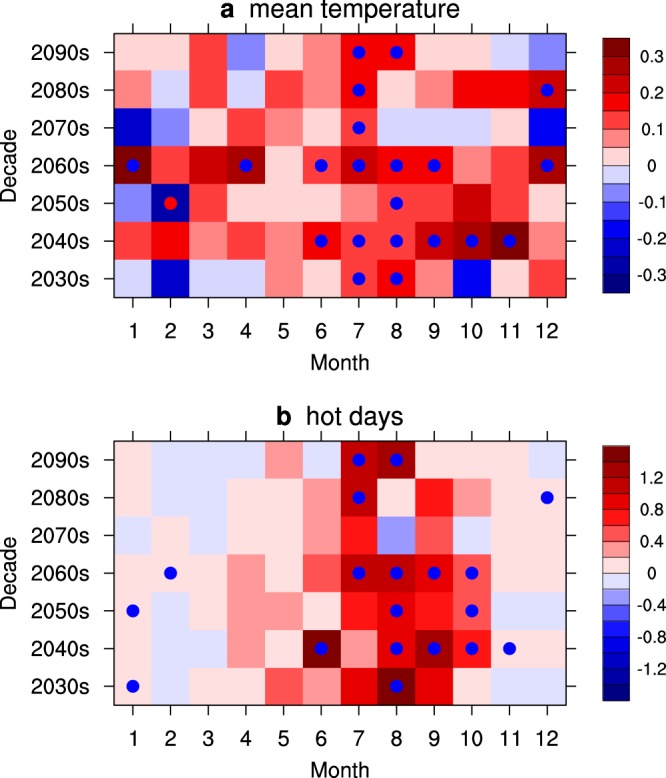
Decade-month section of the area-averaged surface temperature (**a**) and days of extremely hot event (**b**) differences between the OS and NE scenarios over the domain [22–30°N, 100–120°E]. The unit is °C. Dots indicate that the significance level reaches 90%.

This SC warming difference in July will be a significant feature during the last several decades of 21st century. Figure 2a shows the decade-month section of the area-averaged temperature differences in the SC. From the 2070 s to 2090 s, all of the decades exhibit significant differences in July. The differences in other months, except August in the 2090 s, are not significant.

**Figure 3:**
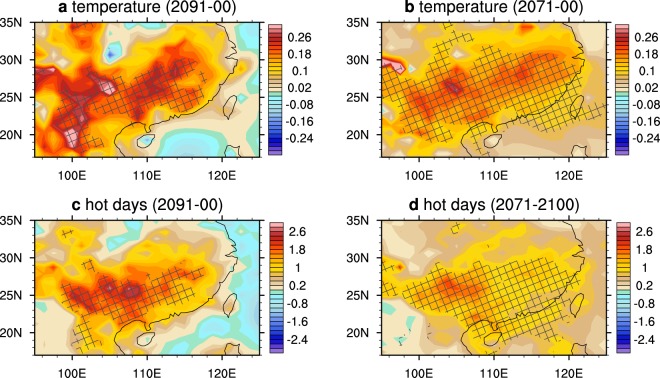
Differences of the surface temperature (unit: °C) and number of extreme hot days (unit: days) between the OS and NE scenarios in July. (**a**) Temperature during 2091–2100; (**b**) temperature during 2071–2100; (**c**) number of extreme hot days during 2091–2100; and (**d**) number of extreme hot days during 2071–2100. Lattices indicate that the significance level of the results reaches 90%.

Not only the area-averaged results but also the patterns of warming differences are significant among ensembles in the SC. In the last decade of the 21^st^ century, the mid-summer in SC under the OS scenario is significantly warmer than that under the NE scenario (Fig. 3a). In some grids, the differences reach 0.3 °C. For the 2071–2100 mean, the SC also has a significantly warmer summer under the OS scenario, except that the differences between the two scenarios are weaker than those in 2091–2100 and that the area that reaches the 90% significance level is wider.

The number of extreme hot days in July in the SC also displays a large discrepancy between the OS and NE scenarios when approaching 2100. Figure 2b shows the decade-month section of the area-averaged differences in the number of extreme hot days in the SC. For the last 3 decades, the maximum increase in the number of extreme hot days in the SC generally occurs in July. The significance levels are slightly lower than those of the mean temperature results (Fig. 2a). In 2005, the mean extreme hot days is 11.5 days in July. During the 2080 s and 2090 s, the mean numbers of extreme hot days are 18.3 and 17.8 days in the NE scenario, respectively, and the mean numbers of extreme hot days are 19.4 and 19.0 days in the OS scenario, respectively. The mean number of extreme hot days increases by 55%-69% in the NE and OS scenarios. During the 2080 s, the mean number of extreme hot days increases by 6.8 days in the NE scenario relative to that in 2005; in the OS scenario, relative to that 2005, the mean number of extreme hot days increases by 7.9 days, which corresponds to a 16% additional increase relative to the increase in the NE scenario. During the 2090 s, the mean number of extreme hot days increases by 6.3 days in the NE scenario relative to that in 2005; in the OS scenario, relative to 2005, the mean number of extreme hot days increases by 7.5 days, corresponding to a 19% additional increase relative to the increase in the NE scenario.

The patterns in the number of extreme hot days in July in the SC between the OS and NE scenarios are also significant. The patterns of the number differences bear some resemblance to those of the July temperatures (Fig. 3). During 2091–2100, the averaged numbers of extreme hot days dominantly increase over the SC. In some grids, the differences reach 2.6 days (Fig. 3c). The area reaching the 90% significance level covers a large part of the SC, except for the southeast coast of China. For the 2071–2100 mean results, the number difference is less than that of the 2091–2100 mean, but the area reaching the 90% significance level is wider (Fig. 3d).

**Figure 4:**
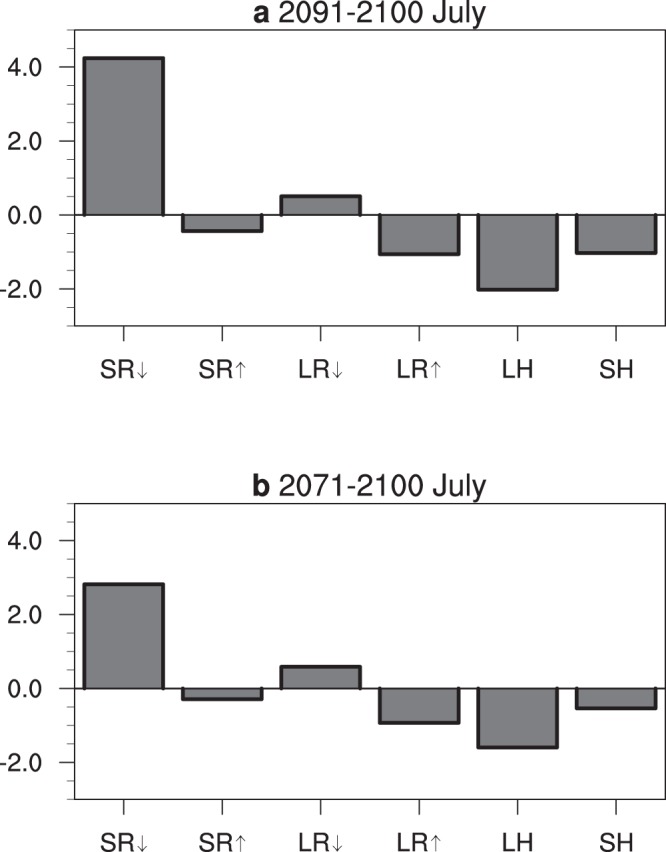
Differences of heat budget terms at the surface over the SC between the OS and NE scenarios. (**a**) The July mean of 2091–2100; and (**b**) the July mean of 2071–2100. The units are W m^−2^. SC is the domain [22–30°N, 100–120°E]. On the x-axis, the radiation terms from left to right are anomalies of downwards solar radiation, upwards solar radiation, downwards longwave radiation, upwards longwave radiation, latent heating flux and sensible heating flux, respectively. Positive and negative, respectively, indicate heat absorption and loss of the surface.

The discrepancy of the heat budget over the SC surface between the two scenarios can help the understanding of the temperature change. The equation for heat budget anomalies is shown in equation (3) in the methods section. In July, during the last decade of the 21^st^ century of the OS scenario, the downwards solar and longwave radiation are both enhanced relative to those of the NE scenario (Fig. 4a). The anomalies of the two are 4.24 W m^−2^ and 0.50 W m^−2^, respectively. The enhancement leads to increased radiation absorption and an increase of the SC surface temperature. The anomalies of an upwards solar radiation, upwards longwave radiation, latent heating and sensible heating to the atmosphere are 0.43 W m^−2^, 1.06 W m^−2^, 2.02 W m^−2^ and 1.03 W m^−2^, respectively. The upwards solar radiation consists of the reflection and scattering of downwards solar radiation, which intensifies as the downwards solar radiation intensifies. The intensities of the other three upwards terms are connected to the surface temperature increase: (1) a surface temperature increase leads to an increase of longwave radiation; (2) an increase in the surface temperature contributes to an anomalous increase in the surface-air temperature difference, which leads to an anomalous intensification of evaporation (latent heating to atmosphere) and sensible heating. The anomalous enhancement of the upward four heating terms cancels the increase of the downwards terms and reaches a balance. The July temperatures in 2071–2100 are similar to those in 2091–2100, but the intensities of the anomalous terms are smaller (Fig. 4b). The anomalies of the downwards solar and longwave radiation are 2.82 W m^−2^ and 0.59 W m^−2^, respectively; the anomalies of the upwards solar radiation, longwave radiation, latent heating and sensible heating to the atmosphere are 0.29 W m^−2^, 1.00 W m^−2^, 1.59 W m^−2^ and 0.54 W m^−2^, respectively.

**Figure 5:**
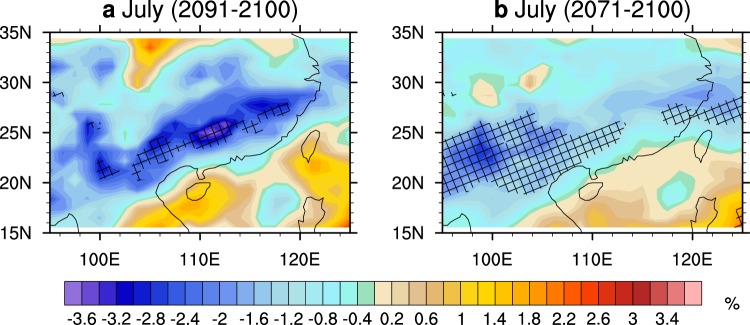
Differences of the vertically integrated mid-level cloud (unit: %) between the OS and NE scenarios in July. (**a**) the average of 2091–2100; (**b**) the average of 2071–2100. Lattices indicate that the significance level of the results reaches 90%.

The downwards solar increase in the SC is mainly due to cloud reduction at the mid-level. The clouds at the low-, mid- and high-levels in CESM1-CAM5 are investigated. We find that the differences of the mid-level clouds between the OS and NE scenarios mostly match the results of downwards solar radiation. During the final 1 to 3 decades, the results exhibit significant differences in mid-level clouds in the SC between the two scenarios (Fig. 5). The difference is larger for the results of the July mean during 2091–2100 and matches the magnitude of downwards solar radiation shown in Fig. 4. Moreover, the difference patterns of the mid-level clouds between the two scenarios share some features with those of the surface temperature change (Fig. 2). The contributions of low-level and high-level clouds are quite small. The differences of both low-level and high-level clouds between the OS and NE scenarios do not match the pattern of the downwards solar increase or the surface temperature increase in the SC (Figs S1 and S2 in the Supplementary materials).

## Summary and Discussion

In this paper, we find that surface warming in the boreal mid-summer in SC may be different if different trajectories of carbon emission are adopted for achieving the Paris Agreement 1.5 °C warming goal. By analyzing the outputs of CESM1-CAM5 under the OS and NE scenarios, it is found that under the OS scenario, the SC mid-summer is significantly warmer than that under the NE scenario in the last 1 or 3 decades of the 21^st^ century. In certain situations, the ratio of additional warming may reach 20% in the OS scenario relative to that of the NE scenario. Moreover, a significant difference is found in the number of extreme hot days in July in the SC between the two scenarios. During the 2090 s, the mean number of extreme hot days increases by 6.3 days in the NE scenario relative to that in 2005; in the OS scenario, an additional increase of 1.2 day is found, corresponding to a 19% increase in the number (6.3 days) in the NE scenario.

The decrease of mid-level clouds in the boreal mid-summer over the SC under the OS scenario relative to that of the NE scenario mainly accounts for the mid-summer warming and extreme hot day differences, because it leads to a rise in downwards solar radiation as well as the surface temperature. The warming surface induces the enhancement of upwards longwave radiation at the surface and latent heat flux into the atmosphere, favoring the balance of the heat budget at the surface.

This paper mainly provides evidence associated with mid-summer warming of the SC under different 1.5 °C warming scenarios. The main cause of the warming difference is the local decrease of mid-level clouds, which may be further influenced by atmospheric circulation, such as the East Asian jet stream^14,15^. How the different trajectories of carbon emissions lead to the discrepancy of mid-level clouds over the SC requires further investigation.

In addition to, if internal variability is taken into account, the above difference of SC surface warming between the NE and OS scenarios may be insignificant. It is found that the internal variability (standard deviations) of the SC mid-summer temperature is greater than the mean difference between the NE and OS scenarios. In the NE scenario, the internal variability (standard deviation) of the 2091–2100 mean July temperature of the SC in the ensembles is from 0.30 to 0.54 °C, and the 2071–2100 means are from 0.31 to 0.50 °C. In the OS scenario, the standard deviations of the 2091–2100 mean July temperature of the SC in the ensembles are from 0.21 to 0.59 °C, and he 2071–2100 means are from 0.27 to 0.49 °C. Among the above cases, the minimum of the standard deviations is 0.21 °C. The mean difference of the SC mid-summer temperature between the NE and OS scenarios is 0.17 °C in 2091–2100 and 0.15 °C in 2071–2100, and both are less than the minimum of the standard deviations (0.21 °C). Thus, the temperature difference over the SC in mid-summer between the two scenarios may not be significant if the internal variability is taken into consideration.

Nevertheless, the above results indicate that different carbon emission strategies for achieving the 1.5 °C warming goal may lead to differences in the multi-year mean response or risk in the regional climate. Policy makers should be cautious when developing climate mitigation strategies.

## Methods

The study in this paper is mainly based on the experiments of CESM1-CAM5. CESM1-CAM5 consists of coupled atmosphere, ocean, land, and sea ice component models. The resolution of all of the model components is approximately 1° horizontally and includes land carbon cycle calculations, diagnostic biogeochemistry calculations for the ocean ecosystem and the atmospheric carbon dioxide cycle^16^. The experiments used in this paper are:

The Large Ensemble experiment^16^. Ensemble 1 of the Large Ensemble experiment is forced by well-mixed greenhouse gases, short-lived gases, aerosols and ozone from 1850–2005. Ensemble 2 is driven by the same forcing as ensemble 1 but from 1920 to 2005; its initialization is 1 January 1920 of ensemble 1, starting with 1-day lagged ocean temperatures. Ensembles 3–35 is driven by the same forcing as ensemble 2; its initialization is 1 January 1920 of ensemble 1 for all of the model components, but with an atmospheric round-off (order of 10^−14^ K) differences in the air temperature.

The 1.5 °C warming (never exceeding) experiment^8^. In this paper, this experiment is named NE for short. This scenario is designed such that the expected multi-year GMT never exceeds 1.5 °C above the pre-industrial levels (where pre-industrial is taken as the 1850–1920 mean) in CESM1-CAM5. Emissions follow RCP8.5 until 2017, after which the carbon emissions rapidly decline, reaching 50 percent of the 2017 levels in one decade by 2027. Combined carbon emissions reach net-zero in 2038. CO_2_ emissions reach a peak net negative level in 2065, with a net flux of −1.8 GtC/yr. After this, negative emission fluxes are reduced, reaching −0.9 GtC/yr by 2100. This experiment includes 10 members.

The 1.5 °C warming (overshoot) experiment^8^. In this paper, this experiment is named OS for short. This scenario is designed such that the expected GMT briefly overshoots before returning to 1.5 °C by 2100 in CESM1-CAM5. Emissions follow RCP8.5 until 2017, after which emissions decline slightly less rapidly than in the NE scenario, such that the emissions are half those of the 2017 levels by 2032. In this scenario, combined carbon emissions reach net-zero in 2046. The overshoot requires a larger late century negative emissions commitment, with a peak net negative flux of −4.0 GtC/yr in 2080. After this, negative emission fluxes are rapidly reduced, reaching −1.0 GtC/yr by 2100. This experiment includes 5 members.

We obtain the above experiment outputs from https://www.earthsystemgrid.org/dataset/ucar.cgd.ccsm4.output.html. The GMT increases of the 3 above experiments are shown in Fig. 1a.

The observational data used to evaluate the reproducibility of CESM1-CAM5 are the 756-meteorological station daily data from the China Meteorological Administration. The data are from 1951–2012. Here, the evaluation is focused on the period of 1976–2005. We used the data from stations whose data contain no missing records from June 1 to August 31 in each year from 1976 to 2005.

The model reproducibility is evaluated based on the *S*-index^17,18^, defined as

1 $$S=\frac{{(1+R)}^{4}}{4{(SDR+\frac{1}{SDR})}^{2}}$$

where *R* is the spatial correlation over the domain [22–30°N, 100–120°E] and *SDR* is the spatial standard deviation in CESM1-CAM5 against that observed over the domain [22–30°N, 100–120°E]. Supposing the evaluated variable is the same in CESM1-CAM5 and the observation, that is, *R* = 1.0 and *SDR* = 1.0, we speculate that the best reproducibility corresponds to *S* = 1.0.

To evaluate the reproducibility of CESM1-CAM5, the simulation used is the Large Ensemble experiment, which was evaluated against the 756-meteorological station data from the China Meteorological Administration. The observational data are interpolated into the grids, which are the same as CESM1-CAM5. The evaluated period is 1976–2005.

We use Student’s *t*-test to test the null hypothesis that the sample means are from the same population. Rejection of the null hypothesis (i.e., acceptance of the alternative hypothesis) indicates that the sample means are from two different populations. The standard deviation used in Student’s *t*-test is among the ensembles, rather than those among years.

To calculate the number of extreme hot days, we perform the following steps: for each grid, we first rank the daily maximum temperatures during 1970–2005 in ascending order and choose the 90^th^ percentile temperature as the threshold; then, the days when the maximum temperature exceeds the threshold are defined as extreme hot days.

At the end of the 21^st^ century, the surface property in the SC may not change fundamentally between the OS and NE scenarios. The heat transfer from surface to deep soil may not show a significant discrepancy between the two scenarios either. Thus, this heat transfer is not taken into consideration in the present study. The equation for the heat budget anomalies at the earth surface is written as

2 $$\frac{\partial T}{\partial t}\approx SR\downarrow +SR\uparrow +LR\downarrow +LR\uparrow +LH+SH$$

where the terms on the right-hand side denote the anomalies of downwards solar radiation, upwards solar radiation, downwards longwave radiation, upwards longwave radiation, latent heating flux and sensible heating flux, respectively. For the 10-yr or 30-yr means, the left-hand side term is approximately equal to 0. Then

3 $$0\approx SR\downarrow +SR\uparrow +LR\downarrow +LR\uparrow +LH+SH$$

## Electronic supplementary material

Supplementary Materials
