# Supplementary material for: Different multi-year mean temperature in mid-summer of South China under different 1.5 °C warming scenarios
Source: Sci Rep. 2018 Sep 14;8:13794. doi: 10.1038/s41598-018-32277-6 (PMC6138630; doi:10.1038/s41598-018-32277-6)
Supplement: Supplementary file 1 — Supplementary Materials [file 41598_2018_32277_MOESM1_ESM.pdf]

Different multi-year mean temperature in mid-summer of South China under different 1.5 °C warming scenarios

Xia Qu[1], Gang Huang[1,2,3*]

[1] State Key Laboratory of Numerical Modeling for Atmospheric Sciences and Geophysical Fluid Dynamics, Institute of Atmospheric Physics, Chinese Academy of Sciences, Beijing 100029, China

[2] Laboratory for Regional Oceanography and Numerical Modeling, Qingdao National Laboratory for Marine Science and Technology, Qingdao 266237, China

[3] University of Chinese Academy of Sciences, Beijing 100049, China

[*]corresponding author, Correspondence and requests for materials should be addressed to Gang Huang (hg@mail.iap.ac.cn).

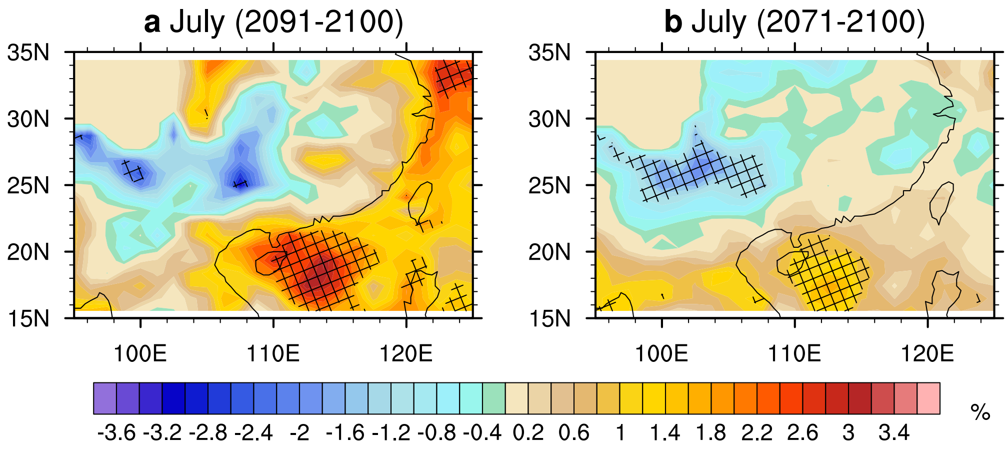

Figure. S1 The differences of vertical-integrated low-level cloud between OS and NE scenarios. (a) July mean during 2091-2100; (b) July mean during 2071-2100. The unit is %. Lattices indicate the significance level of the rainfall results reaching 90%.

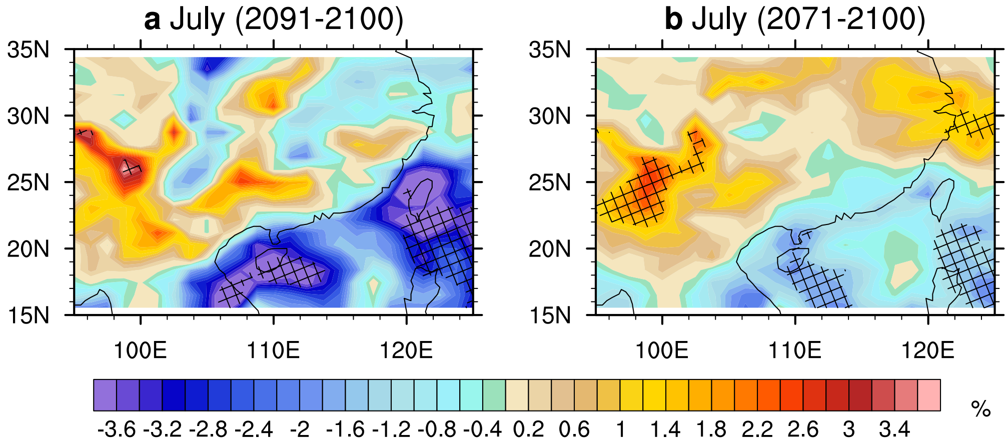

Figure. S2 The same as Fig. S1, except for vertical integrated high-level cloud.
